# Impulsivity influences betting under stress in laboratory gambling

**DOI:** 10.1038/s41598-017-10745-9

**Published:** 2017-09-06

**Authors:** Natale Canale, Enrico Rubaltelli, Alessio Vieno, Andrea Pittarello, Joël Billieux

**Affiliations:** 1 Department of Developmental and Social Psychology, University of Padova, Padova, Italy; 2 University of Groningen, Groningen, The Netherlands; 3 Addictive and Compulsive Behaviour Lab (ACB-Lab). Institute for Health and Behaviour, Integrative Research Unit on Social and Individual Development (INSIDE), University of Luxembourg, Esch-sur-Alzette, Luxembourg; 4 Addiction Division, Department of Mental Health and Psychiatry, University Hospitals of Geneva, Geneva, Switzerland

## Abstract

Although recent research suggests that acute stress influences subsequent decision-making under ambiguity, less is known about the role of personality variables in this relationship. This study tested whether impulsivity traits and acute stress differentially influence the way in which a prior feedback is incorporated into further decisions involving ambiguity. Sixty college students (50% male; aged 18–25 years) were randomly assigned to a stress versus a non-stress condition before completing a laboratory gambling task. The results revealed that independently of the stress condition, subjects behaved as if the odds of winning increase after a single loss. Additionally, stress effects varied as a function of impulsivity traits. Individuals who lacked perseverance (i.e., had difficulty focusing on a difficult or boring task) gambled more after experiencing a loss in the stress condition than did those in the control condition. The present study supports that impulsivity traits can explain the differential effect of stress on the relationship between prior feedback and choices made under ambiguity.

## Introduction

Everyday decision-making (e.g., choosing the correct alternatives in an exam or making an appropriate decision in an emergency situation) is often made under stressful conditions, in which predictions of ambiguous options can be altered by stress^1^. According to a recent meta-analysis^2^, two types of mechanisms may explain how acute stress influences subsequent decision-making in situations of ambiguity. First, acute stress tends to increase reliance on immediate and potentially high rewards at the cost of considering potential delayed losses^3^. From such a perspective, increased reward seeking is supposed to be the underlying mechanism for poor performance. Second, the excessive release of dopamine, noradrenaline, and cortisol related to stress impairs executive control (for a review, see Hermans *et al*.^4^), leading people to rushed and unsystematic decision-making characterized by a tendency to elude the available options (for a review, see Janis & Mann^5^). Another candidate factor that has been linked to biased or maladaptive decision-making under stress refers to personality variables. Actually, little attention has been directing toward the potential moderating role of personality traits in disadvantageous decision-making according to a recent meta-analysis^2^. The current study thus aimed to address this gap in the literature.

In ambiguous situations in which the probability of an outcome is largely unknown, individuals have to infer these probabilities by relying on previous feedbacks associated with similar decisions made in the past. This feedback can be used to rely on adaptive strategies during decision-making, such as balancing choices based on reinforcement schedules or outcome probabilities. In other words, adapted decision-making generally requires inferring the expected reward values of each option based on the consequences of previous choices and favouring the options with the higher expected value. According to the influential somatic marker hypothesis^6^, when faced with a context of ambiguity, individuals unconsciously take into account (or do not) previous positive and negative outcomes of their actions on the basis of “somatic markers” (e.g., feelings and hunches experienced after receiving feedback)^7^. Somatic markers are generally considered anticipatory emotional reactions processed implicitly and triggered by a situation in which a decision has to be made. The somatic marker hypothesis also states that the connection between somatic marker (or feedback) processing and decision-making is susceptible to be influenced by individual differences in reinforcement sensitivity (e.g., a heightened sensitivity to rewards and/or punishments), especially when decisions must be made in situations of ambiguity in which no other clues besides feedback are available^8^. More recently, a revised model of decision-making under objective risk conditions^9^ proposed, in line with classic dual process models^10, 11^, that information about the decision situation is processed via two distinct modes: an impulsive system (involving emotional reactions, conditioning, and somatic activity) and a reflective system (involving working memory, executive functions, and reasoning). External influences, such as stress, may interfere with the processes underlying the reflective system, which are necessary for the normative calculation of probabilities and/or monitoring of feedback.

Research suggests that stress-induced changes may alter both executive functioning and emotional feedback processing^2^. Stress leads to an increase in dopaminergic activity through the release of the stress hormone cortisol^12^, and this elevated dopamine response has been suggested to influence reward prediction and feedback learning^13^. More precisely, individuals experiencing stress have been found to focus on more immediate gratification and tend to have difficulties in delaying rewards^3^. In addition, an increase of dopamine level under acute stress is responsible for a decrease in the willingness to avoid potential losses in laboratory decision-making tasks. For example, in a study using a task measuring decision-making when the odds are known (game of dice task^14^), it appeared that individuals are susceptible to stress-induced disadvantageous decision-making (i.e., they take more risks and focus on potential high short-term rewards and neglect potential higher long-term punishments).

Decision-making under ambiguity is also influenced by erroneous beliefs about randomness. The classic economy theory states that rational agents should not consider the outcome of past experiences on decisions to take a new gamble^15^. According to this rationalist perspective, each new decision to gamble should be considered an independent event. However, it is established that individuals are actually influenced by past experiences (recent outcomes) when making choices with ambiguous results. For example, real-life gamblers regularly display the “gambler’s fallacy” phenomenon^16^, or the tendency to respond to losses by increasing one’s bet, which reflects an inability to acknowledge the independence of turns. Gambling tasks have regularly been used to examine decision-making under ambiguity that require the processing of feedback of previous decisions. In this vein, FeldmanHall *et al*.^17^ found that in a gambling task (unknown probabilities of possible outcomes) in which participants are required to choose to gamble between 0€ and 10€, individuals gambled more after experiencing a loss than a win. The authors interpreted this finding by assuming that participants behave as if the odds of winning increase after a single and/or run (e.g., two) of loss outcomes. Such behaviour seems to reflect a distorted cognition that is often present in people displaying disordered gambling behaviours^16^. Furthermore, FeldmanHall *et al*.’s experiment^17^ also showed that acute stress, induced by using the cold pressor test (CPT^18^), did not impact the use of past experiences for subsequent decision-making under ambiguity among participants. However, it remains unclear whether individual differences (e.g., personality traits) could actually help in explaining the differential effect of stress in the relationship between prior feedback and choices made under ambiguity.

In reviewing the literature on the effects of stress on decisions under ambiguity, little attention has been devoted to personality variables, which have been found to moderate the effect of stress on decision-making (see Starcke & Brand^2^ for a recent meta-analysis). More specifically, the few studies that have assessed the effect of personality traits on decisions made under stress reported that trait anxiety interacts with acute stress in predicting risky decisions^19–21^. Nevertheless, one aspect of personality susceptible to playing a pivotal role in decision-making under stress is impulsivity (see Starcke & Brande^22^ for a review). Impulsivity, which is a construct included in almost all major personality models^23^, is known to influence a wide range of common behaviours (e.g., eating, consumer behaviours) and thus constitutes an important aspect of daily life decision-making. Moreover, impulsivity is also consistently related to gambling disorders (see MacLaren *et al*.^24^ for a review). On a broader level, impulsivity can be considered a trans-diagnostic etiological factor involved in the aetiology of a wide range of mental and neurological disorders characterized by decision-making impairment^25^. It is currently acknowledged that impulsivity is an “umbrella” construct which includes several distinct traits^23, 26^. In the last decade, the UPPS (Urgency-Premeditation-Perseverance-Sensation Seeking) Impulsive Behavior Scale has become one of the most used instruments to assess the multifaceted nature of impulsivity^23, 27^, especially in the gambling field^28^. The UPPS model^23, 27^ distinguishes between five specific impulsivity components: (i) negative urgency, the tendency to act rashly when experiencing intense negative emotions; (ii) premeditation, the tendency to take into account the consequences of an act before engaging in that act; (iii) perseverance, the ability to remain focused on a task that may be boring and/or difficult; (iv) sensation-seeking, the tendency to enjoy and pursue activities that are exciting and openness to trying new experiences; and (v) positive urgency, the tendency to act rashly when experiencing intense positive emotions. Some studies reported relationships between the facets of the UPPS model and the performance in the Iowa gambling task (IGT), a classic paradigm measuring decision-making under ambiguity^14^. More specifically, a proneness for disadvantageous choices in IGT performance was related to a heightened level of negative urgency^29–31^ and a reduced level of premeditation^32^ in healthy individuals. As negative urgency is conceptualized as a type of emotion laden impulsivity and based on existing evidence having linked this impulsivity facet to poorer performances in the IGT, it is reasonable to expect that individual differences in negative urgency will influence decision-making under stress. Moreover, a corpus of behavioural and neuroimaging data suggests that heightened negative urgency could be at least partly due to prepotent response inhibition impairment^29, 33^. Although these results concern negative urgency (these studies did not assess positive urgency), it is reasonable to assume that processes underlying positive and negative urgency are at least partly similar, as emphasized in a recent meta-analysis showing very similar associations between both positive and negative urgency and psychopathological symptoms^34^. Accordingly, and taking into account the impairing nature of emotional arousal on the efficacy of executive functioning^35, 36^, it is likely that individuals with elevated positive and/or negative urgency levels will be more prompt to display hazardous decision-making in stressful situations. In a recent study conducted by Wise and colleagues^37^, it was shown that risky decisions under stress – measured using the balloon analogue risk task (BART^38^) – were affected by complex interactions between the UPPS-P impulsivity traits and gender. They found that stress effects varied as a function of gender and impulsivity traits (negative urgency, positive urgency and lack of perseverance). In particular, Wise and colleagues^37^ reported that stressed women with low perseverance made fewer risky decisions, whereas stressed men with higher perseverance made more risky decisions.

Thus, based on existing evidence, it is likely that impulsivity traits – negative and positive urgency, lack of perseverance and lack of premeditation – may moderate the effect of stress on decision-making under ambiguity. It is conceivable that acute stress influences subsequent decision-making under ambiguity in high emotion contexts (e.g., negative urgency). According to limited cognitive resource theories^39^, it is likely that under acute stress, cognitive resources are directed to emotion regulation and take away from the inhibitory processes that are required to restrain from impulsive choices. An alternative explanation would be that limited cognitive resources caused by stress^40^, such as attentional difficulties, may make individuals characterized by low perseverance particularly vulnerable toward disadvantageous decisions.

Substantial evidence indicated that stress, past experiences (i.e., the influence of prior feedback), and impulsivity traits conjointly affect decision-making processes. However, no studies to date have attempted to disentangle the interplay of these various factors in an experimental design. Therefore, the present study aimed to extend previous research by differentiating the mechanisms of decision-making under acute stress as a function of individual differences in impulsivity traits. More specifically, this study tested whether impulsivity traits and acute stress differentially influence the way in which prior feedback is incorporated into further decisions involving ambiguity. Previous studies have found that the nature of feedback can promote both conservative and risky decisions in the condition of decision under risk (see Schiebener and Brand for a recent review^9^). Schiebener and Brand^9^ suggested that these differences may be systematically related to the feedback structure (e.g., positive feedback versus negative feedback), such that the feedback occurring after a decision is used to (i) check the outcome of the current decision-making strategy and (ii) monitor and eventually revise the applied strategy^41^. In the current study, we investigated whether individuals rely on immediate feedback related to a previous decision for making more advantageous decisions. For this purpose, we tested and compared two models that included decision-making under ambiguity, stress, impulsivity, and feedback. The difference between the two models is the way in which the feedback was coded. More specifically, in model 1, feedback was coded as −1 for a loss in the gambling task, as 0 for a trial in which the participant chose not to gamble, and as 1 for a win in the gambling task (two specific contrasts are analysed in model 1: win relative to loss, and no feedback relative to loss), while in model 2 it was coded as 0 for no feedback and as 1 for a loss/win (a single contrast is analysed in model 2: no feedback relative to loss/win).

Here, we defined stress as a physiological response of the organism that occurs whenever a demand exceeds regulatory capacity, particularly in unpredictable and uncontrollable situations^42^. A classic paradigm used to induce stress experimentally in the laboratory is the CPT^18^, in which participants must immerse one hand in a basin of icy water. By directly manipulating acute stress with the CPT – indexed by subjective parameters of stressors^43^, we conducted an experiment designed to test whether impulsivity traits and acute stress would differentially influence the way in which prior feedback is incorporated into decisions involving ambiguity. The present experiment was conducted with the gambling task used by FeldmanHall *et al*.^17^ to assess decisions under ambiguity (the source of ambiguity is the fact the probability of winning gambles are unknown to participants). In this laboratory gamble task, participants were required to choose in a series of trials whether to gamble an amount between 0€ and 10€. The task required the participant to make 36 choices (36 trials), and in each trial, participants could win or lose their money. After each decision, subjects were informed about the outcome (i.e., feedback is provided).

Consistent with the theoretical backgrounds reviewed, we expect that, in a stress condition, participants characterized by elevated negative urgency will gamble more after having experienced a loss in a previous trial than those with higher negative urgency in a non-stress condition, as this facet of impulsivity has been postulated to promote the involvement in rushed action aiming at relieving negative affect^27^. Participants in the stress condition and characterized by higher negative urgency should display reduced executive control that overrides emotion-induced risk taking propensities in favour of executive control, decreasing the probability of making advantageous decision-making^9^. Furthermore, we also hypothesized that the probability of choosing to gamble after a loss will be higher in stressed subjects with lower levels of premeditation and perseverance, as these cognitive dimensions of impulsivity have respectively been related to poor decision-making abilities^44^ and impairment in resistance to proactive interference (i.e., the ability to inhibit previous information that is no longer relevant)^45, 46^. Stress combined with lower perseverance/premeditation should strengthen the impulsive system, which in turn is susceptible to interfere with the controlled extraction of information, deliberation, or planning (reflective system), leading participants to act against their better knowledge (e.g., gambled more after experiencing a loss in the previous trial)^9^. Our interest focused on negative urgency, lack of premeditation and lack of perseverance due to relationships with risky decisions under stress^37^ and proneness for risky choices during the IGT^29, 32^. Additionally, positive urgency was not considered because it is conceptually very similar to negative urgency (see Berg *et al*.^34^ for a recent meta-analysis) and the stressor used in the current study is known to elicit negative affect^19^, implying that focusing only on negative urgency is more relevant.

## Methods

### Participants

Sixty college students (age 18–25 years) were recruited from the University of Padova. The sample size was determined based on past work using the same task^17^. Participants were randomly assigned to be in either the stress condition (n = 30; 15 males; mean age = 21.88 years, SD = 0.34) or non-stress condition (n = 30; 15 males; mean age = 21.77 years, SD = 0.34). Participants provided written informed consent and were paid an initial €5. They also received additional monetary compensation based on the result of one randomly selected trial from the gambling task. The institutional review board at the University of Padova gave ethical approval for the study. Ethical principles were carried out in accordance with the Declaration of Helsinki.

### Procedure

To control for circadian rhythms and stress induced by travel, the experiment was always conducted between 1:00 pm to 5:00 pm, as stress levels have been shown to fluctuate throughout the day^47^. Participants were scheduled for a one-hour experimental session after having provided informed consent. At the beginning of the experiment, participants were invited to complete self-report questionnaires (demographic questionnaire and perceived stress scale). Next, participants in the stress condition were subjected to a stress manipulation (CPT), whereas those in the non-stress condition were engaged in a non-stress manipulation. To assess the stress induction, participants completed subjective rating of stress, pain and unpleasantness^43^. The gambling task was administrated directly following the stress-induction condition. The experimenter started the computer program and asked the participant to follow the instructions on the screen (see FeldmanHall *et al*.,^17^ for more details about verbal and visual instructions). Upon completion of the gambling task, the short UPPS-P scale^48^ and the Trait Emotional Intelligence Questionnaire^49^ were administered. Data on trait emotional intelligence (not related to the current paper) will be presented elsewhere. Participants were debriefed and paid at the end of the experiment.

### Stress Induction

The CPT was selected for experimentally inducing acute stress, as it is known to have good reliability and validity^50, 51^ and is not characterized by lasting psychological effects (e.g., stress, mood and nervousness) that have been related to other types of laboratory stressors^52^. Participants first held their non-dominant arm (to the elbow) in room temperature water for 2 minutes to ensure an equal starting point^53^. They then transferred their arm into ice water (0–4 °C) for as long as possible up to 2 min^53, 54^. A no-stress control condition required immersion of the participants’ dominant hand in room-temperature water (32–35 °C) for 2 minutes.

### Gambling Task

**Figure 1:**
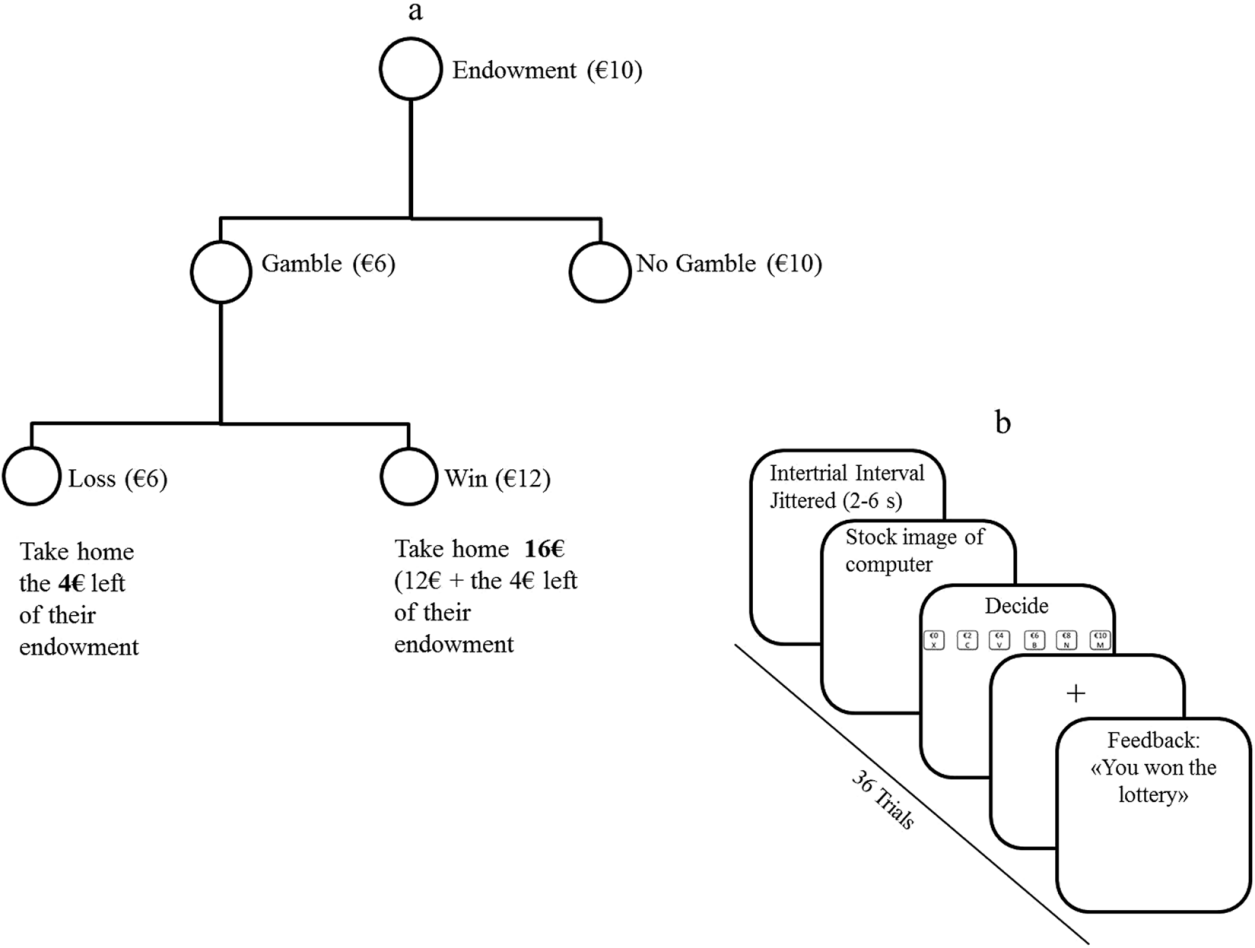
The gambling task^17^: (**a**) example of gambling decisions and (**b**) schematic of one trial of the task.

In each trial of the gambling task^17^, subjects were endowed with €10 (placed on the table) and decided whether to gamble between €0 and €10 of their €10 endowment in increments of €2. If they decided to gamble and lost, they would lose their money. In contrast, if they decided to gamble and won the lottery, they would double their money. For example, subjects decided to gamble €6 of the €10. In one scenario there is the chance to win, double their money (€12), and take home €16 (€12 + the €4 left of their endowment). In the other scenario, they would lose their investment of €6 and take home €4 (Fig. 1a). Subjects completed four practice trials before starting the gambling task. The task consists of 36 trials. During each trial, subjects were presented with stock image of a computer and were given unlimited time to make their gambling decision (Fig. 1b). After their decision, subjects were presented with a fixation cross (jittered duration, 2–6 s) and then either negative feedback (“You lost the lottery”) or positive feedback (“You won the lottery”) for 3 s. Following the feedback, there was an inter-trial interval (jittered duration, 2–6 s). The trials were presented in random order. Subjects did not receive any information about the probability of winning or losing a gamble, implying that the task measures a situation of decision-making under ambiguity. They were instructed that all gambles were independent. To ensure that subjects believed that, we probed their beliefs about the independence of their gambles during a funnel debriefing after testing. No participants indicated any suspicions regarding the delivered outcomes (wins or losses). The outcome variable from this task is subjects’ choices to gamble (how much money subjects gambled from €0 to €10). One potential predictor from this task is feedback on the previous trial or feedback (See “Statistical Analyses” section for further details).

### Self-report questionnaires

At the beginning of the experiment, participants completed the perceived stress scale (PSS), which assesses the degree to which life events are appraised as stressful^55^ (Italian translation: Fossati^56^). The PSS contains 10 items rated on a 5-point Likert scale, ranging from 0 (*never*) to 4 (*very frequently*). Higher scores reflect higher levels of perceived stress in response to stressful situations. Internal consistency for the PPS was adequate in the present sample (α = 0.75, 95% CI [0.65, 0.84]). As the interaction between the trait of perceived stress and acute stress could have a significant effect on deliberative/decision-making processes rates (e.g., delayed discounting^19^), participants in the stress-induction condition were compared with those in the control condition for the trait of perceived stress at the baseline to isolate the effect of acute stress on choices. Demographic information was also collected (gender, age and school attendance). In the present research, the PPS and demographic characteristics were used as control variables.

To validate that stress induction was effective, we measured subjective parameters of stressors^38^. Directly after the participants took their hands out of the cold or room-temperature water, ratings of stress, pain and unpleasantness were assessed. Subjects rated separately on scales ranging from 0 (*not at all*) to 100 (*very much*) in 10-point increments: first, how stressful the hand immersion was, then how unpleasant it was, and then how painful it was.

Directly after having completed the gambling task, participants completed the short UPPS-P^48^ (Italian version: D’Orta *et al*.^57^). The UPPS-P is a 20-item scale assessing five impulsivity traits (four items per dimension) including negative urgency, lack of premeditation, lack of perseverance, sensation seeking and positive urgency. All items are scored on a Likert scale from 1 (*strongly agree*) to 4 (*strongly disagree*). All of these scales demonstrated adequate internal consistency in the present sample: negative urgency (α = 0.79, 95% CI [0.70, 0.87]), (lack of) premeditation (α = 0.77, 95% CI [0.66, 0.85]) and (lack of) perseverance (α = 0.88, 95% CI [0.82, 0.92]). For each trait, higher scores indicate a higher level of impulsivity.

### Statistical Analyses

The statistical analyses were performed in R. Specifically, we used the lme4^58^ and lmerTest packages^59^ in R to run a series of linear mixed-effects models (LMMs; see the results below). Maximum likelihood t- and F-tests were conducted by using Satterthwaite approximations for pooled degrees of freedom, using the lmerTest package^59^. To explore the effects of stress and impulsivity traits on individuals’ ability to incorporate feedback, we tested and compared two LMMs that included subjects’ choice to gamble (dependent variable; how much money subjects gambled, from 0 to 10€) and all parameters (feedback, condition, impulsivity traits, and their interactions); all parameters were included in the model as independent variables (fixed effects). The only difference between the two models was the way in which feedback was coded (see the results below). The two LMMs were compared, respectively, with a null model, which included only the intercepts and no predictors. The degrees of freedom (df) for all the models correspond to the number of parameters included (i.e., main effects, two, and three way interactions) plus one parameter for each random effect, one for the intercept, and one for the variance associated with the random effect (i.e., the null model consists of three degree-of-freedom: one for the intercept, one for the random effect of subject, and one for the variance associated with it). Finally, in the two LMMs we included time as a control variable and subject as a random effect. The within-subjects predictor was feedback received on the previous trial, while the between-subjects predictor was condition. Lagged feedback (on the previous trial t − 1) was coded as −1 for a loss in the gambling task, as 0 for a trial in which the participant chose not to gamble, and as 1 for a win in the gambling task (in model 1, M1), while it was coded as 0 for no feedback and as 1 for a loss/win (in model 2, M2). (See Supplemental Materials for detailed results of alternative models: A learning model in which feedback is coded to take into account the combined outcome of the previous three trials (e.g., two wins and one loss), and another model which includes the amount won or lost in the previous trial as a predictor). The condition was coded as −1 for control and 1 for stress. Time was coded as 1 (trial 1 to 12), 2 (trial 13 to 24), and 3 (trial 25 to 36).

## Results

### Preliminary analyses

To allow interpretation of the regression analyses conducted, we had to ascertain that (i) there was no sign of multicollinearity; (ii) there were no differences between stress condition and control with regard to impulsivity traits; and (iii) the CPT had induced stress, whereas the control condition had not. With regard to the check for collinearity, the magnitude of correlation coefficients was relatively modest, ranging from −0.06 to 0.47. To control for the presence of multicollinearity, we computed the variance inflation factor (VIF), which shows how much the variance of the coefficient estimate is inflated by multicollinearity. VIF values over 2.5 are considered problematic for multicollinearity^60^. The VIF values in the present study ranged between 1.02 to 1.69. Thus, no multicollinearity existed.

In addition, the two groups did not differ significantly in their ratings of impulsivity (see Table 1).

**Table 1:** Impulsivity traits between conditions.

	Control Condition Room-temperature water (n = 30)	Stress Condition Cold Pressor Test (n = 30)	F_(1, 58)_	p	ƞ^2^
*Impulsivity traits*					
Lack of Premeditation	7.77(0.36)	6.80(0.34)	3.62	0.06	
Negative Urgency	8.93(0.49)	8.26(0.48)	0.90	0.34	
Lack of Perseverance	7.10(0.45)	7.00(0.45)	0.02	0.88	

Finally, with regard to stress induction, subjective parameters support that CPT is a reliable stressor (see Table 2). Participants experienced significantly more stress, pain and displeasure (stress F_(1, 58)_ = 46.11, *p* < 0.001, ƞ^2 = ^0.44; pain, F_(1, 58)_ = 155.62, *p* < 0.001, ƞ^2^ = 0.73, unpleasant, F_(1, 58)_ = 141.16, *p* < 0.001, ƞ^2^ = 0.71) in the stress condition than in the control condition. The stressful rating was strongly correlated with the painful (*r* = 0.75, p < 0.001) and unpleasant (*r* = 0.81, p < 0.001) ratings. In addition, the PSS score (administered before introducing stress manipulation) did not significantly vary by group (F_(1, 58)_ = 0.05, *p* = 0.94), implying that participants in these groups exhibited similar perceived stress in response to stressful situations (Table 2).

**Table 2:** Subjective stress ratings and Perceived Stress Scale.

	Control Condition Room-temperature water (n = 30)	Stress Condition Cold Pressor Test (n = 30)	F_(1, 58)_	p	ƞ^2^
Subjective Stress Ratings					
Unpleasant	5.33(1.64)	60.33(4.32)	141.16	<0.001	0.71
Stressful	7.67(2.33)	42.33(4.54)	46.11	<0.001	0.44
Painful	3.33(2.10)	60.33(4.05)	155.62	<0.001	0.73
Perceived Stress Scale (PSS)*	18.33(1.06)	18.20(1.54)	0.005	0.94	0.000

* *Administered before the stress manipulation*.

With regard to the money gambled in each condition, subjects gambled approximately the same amount of

money in the control condition and in the stress condition, while participants gambled at a higher rate in the

stress condition than they did in the control condition (See the Supplemental Material for details; Money

gambled for condition and Choice rate in the lottery game).

### Effects of acute stress and impulsivity on sensitivity to feedback: trial-by-trial analysis

To test how subjects with higher impulsivity incorporate prior feedback into decision-making under stress, we tested and compared the following models: (a) the null model with intercept only and no predictors (M0); (b) the model with stress, impulsivity traits, feedback, and their interactions, where feedback was coded as −1 for a loss, 0 for no feedback, and 1 for a win (M1); (c) the model with stress, impulsivity traits, and feedback and their interactions, where feedback was coded as 0 for no feedback and 1 for a loss/win (M2). The difference between M1 and M2 was basically the way in which the feedback was coded. To compare the models, we performed the likelihood ratio test and took into consideration the Bayesian information criterion^61^. Table 3 shows the results for the model comparison. ΔBIC refers to the differences between the null model (M0) and the other models (M1 and M2); a positive ΔBIC value indicates that a model (M1 or M2) is better than the null model. Bayes factor (BF) approximations were calculated by using the formula exp(ΔBIC/2)^62^. BF approximations were used to compare the relative evidence for different models. For example, a BF value of 4 indicates that one model is four times more likely than the null model (M0). In summary, the higher the ΔBIC and BF approximations, the more likely the model is in comparison to the null model. As can be seen in Table 3, M1 showed a better fit than M2.

**Table 3:** Model comparisons.

	Model *df*	Chisq	*p*	AIC	BIC	ΔBIC	Approx. BF
M0	3			10380	10397		
M1	19	165.56	<0.001	10246	10353	43.17	>10,000
M2	19	90.01	<0.001	10322	10429	−32.32	<10,000

M0 = null model; M1 = model with stress, impulsivity traits and feedback and their interactions (feedback = −1 for a loss, 0 for no feedback and 1 for a win); M2 = model with stress, impulsivity traits and feedback and their interactions (feedback = 0 for no feedback and 1 for a loss/win); df, degree of freedom; Chisq, chi-squared ; p, probability value; AIC, Akaike information criterion; BIC, Bayesian information criterion; ΔBIC, differences between the null model (M0) and other models (M1, M2); Approx. BF, Bayes factor approximation, exp(ΔBIC/2).

**Figure 2:**
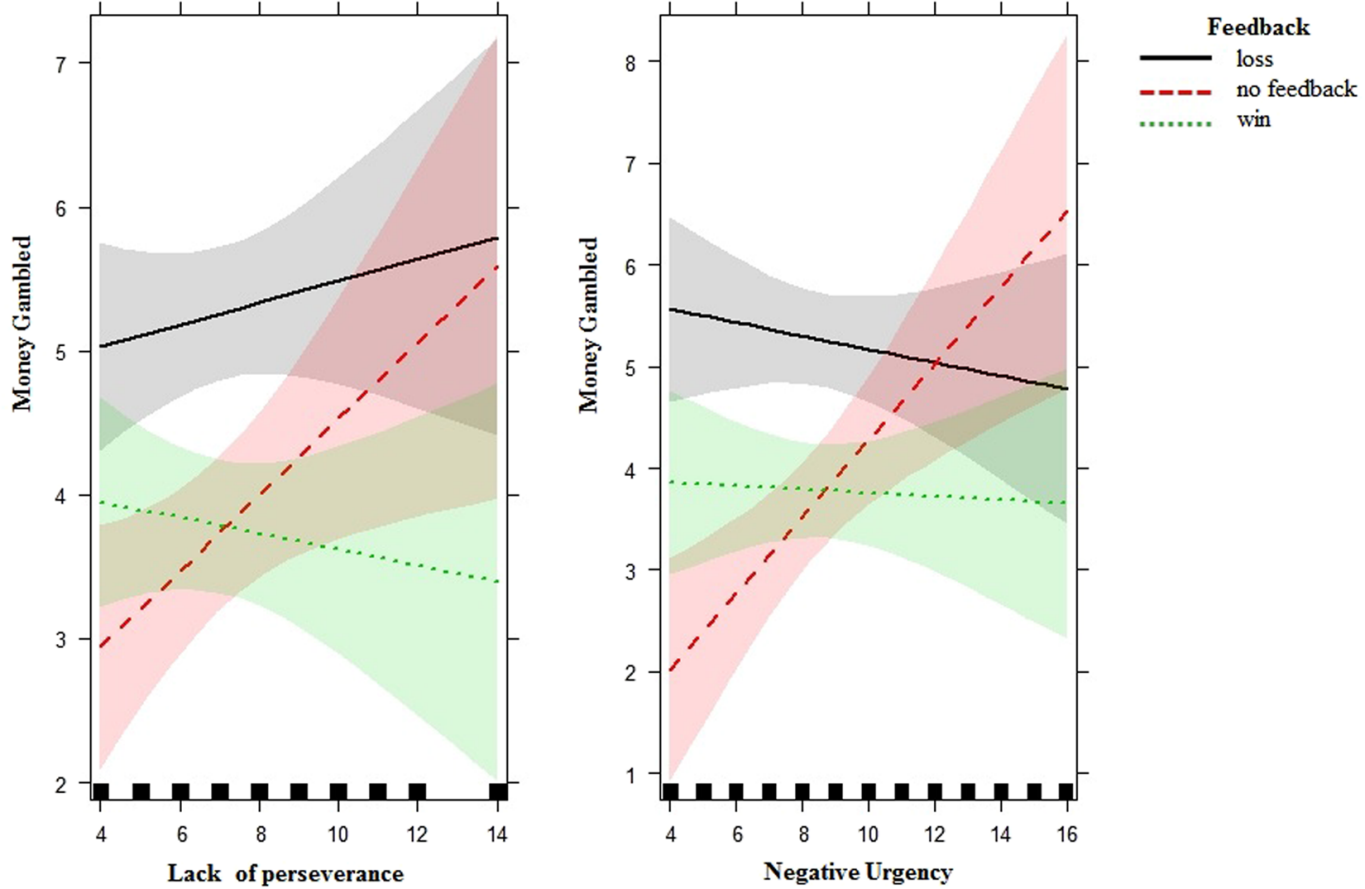
Interaction plot for lack of perseverance/negative urgency and feedback in relation to the amount of money gambled. feedback = feedback on the previous trial (win = a win in the gambling task, loss = a loss in the gambling task, no feedback = the subject chose not to gamble). Money gambled = subjects’ choices to gamble (i.e., how much money subjects gambled from €0 to €10). Confidence bands of 95% are presented in grey/red/green.

**Figure 3:**
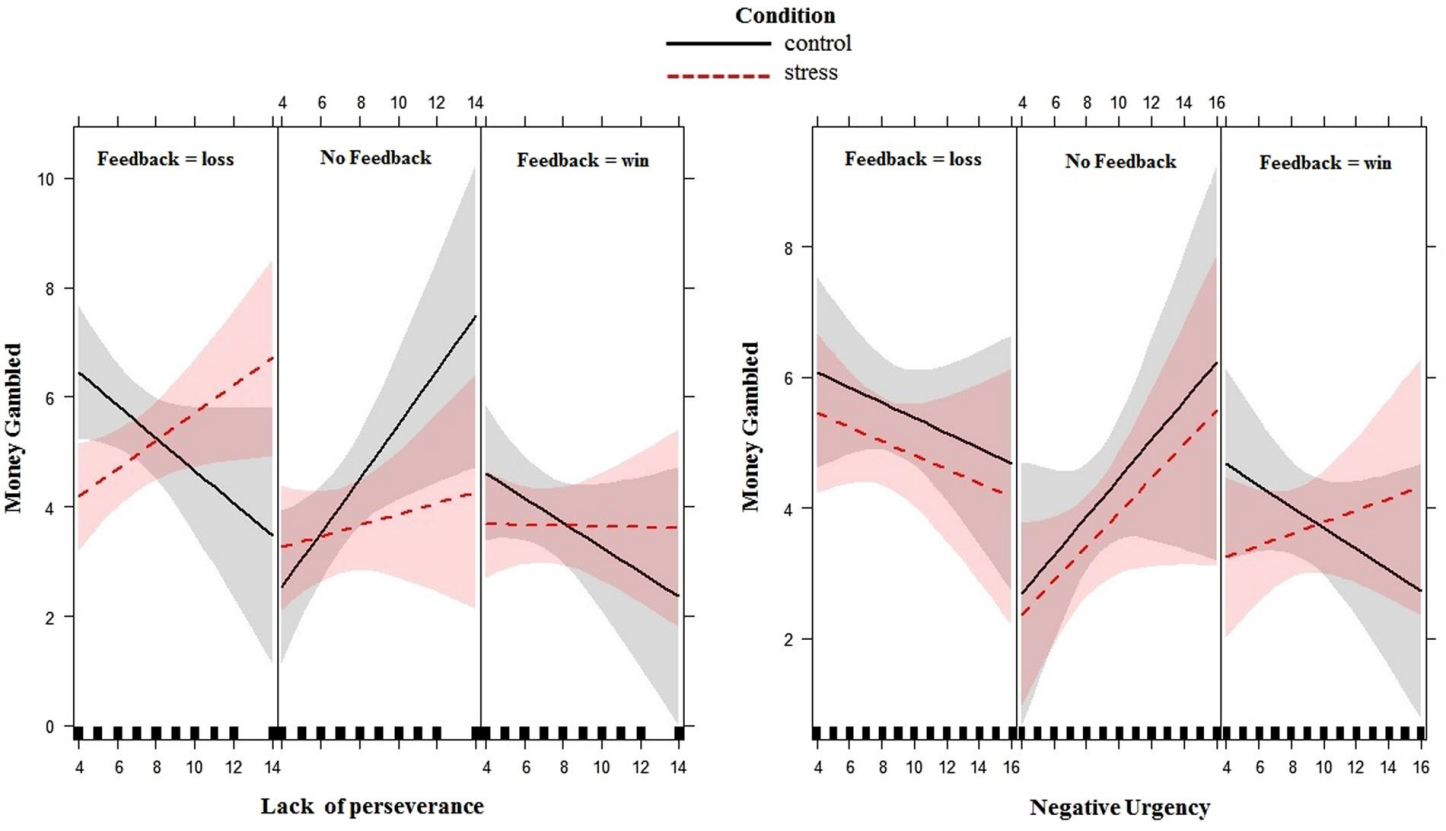
Interaction plot for impulsivity traits, condition and feedback in relation to the amount of money gambled. Money = gambled subjects’ choices to gamble (i.e., how much money subjects gambled from €0 to €10). Feedback = feedback on the previous trial (win = a win in the gambling task, loss = a loss in the gambling task, no feedback = the subject chose not to gamble). Confidence bands of 95% are presented in grey/red.

Table 4 shows the mixed-effects model (M1), while Table 5 displays the planned comparisons (the effects for each level of the categorical variables and their interactions with impulsivity traits). With regard to M1, stress and the three impulsivity traits (lack of perseverance, lack of premeditation and negative urgency) were not associated with the amount of money gambled (Table 4). However, results revealed a main effect of feedback (χ^2^_(2)_ = 158.60; p < 0.001) and time (χ^2^_(1)_ = 5.87; p = 0.02). Participants gambled less during the final trials (B = −0.17, t = −2.43, p = 0.02) (Table 5). A closer inspection of the results (Table 5) indicates that subjects gambled more after experiencing a loss than after deciding not to gamble in the previous trial (B = −11.17, t = −6.48, p < 0.001). There were also interactive effects of feedback × lack of perseverance (χ^2^_(2)_ = 12.29; p = 0.002) and feedback × negative urgency (χ^2^_(2)_ = 15.35; p < 0.001) (Table 4). To probe the interaction effects, we interpreted significant interactions (p < 0.05) by using contrasts^63^. Results showed that individuals with higher perseverance gambled less after deciding not to gamble than after experiencing a loss (B = −0.18, χ^2^_(1)_ = 5.40, p = 0.02), while those with lower perseverance gambled more after experiencing a loss (B = 0.13, χ^2^_(1)_ = 6.54, p = 0.02) and deciding not to gamble (B = 0.32, χ^2^_(1)_ = 15.46, p < 0.001) than after receiving a win (see Fig. 2). With regard to negative urgency (see Fig. 2), individuals with higher negative urgency gambled more after deciding not to gamble than after receiving a loss (B = −0.44, χ^2^_(1)_ = 25.23, p < 0.001) and a win (B = 0.39, χ^2^_(1)_ = 20.15, p < 0.001). Finally, we found two significant three-way interactions: Stress × Feedback × Negative urgency (χ^2^_(2)_ = 8.24; p = 0.02) and Stress × Feedback × Lack of perseverance (χ^2^_(2)_ = 26.28; p < 0.001) (Table 4). Results showed that the effect of lack of perseverance in interaction with stress was significant only for a loss as previous feedback (B = −0.42, χ^2^_(1)_ = 3.94, p = 0.04), but not for a win (p = 0.78) or no feedback (p = 0.64). As can be seen in Fig. 3, individuals in the stress condition with lower perseverance gambled more after experiencing a loss than did those in the control condition. Acute stress appeared to influence how prior feedback (e.g., a loss in the previous trial) was incorporated into decisions involving uncertainty for subjects with lower perseverance. No significant interaction contrasts for the three-way Stress × Feedback × Negative urgency interaction were found.

**Table 4:** Results of the linear mixed-effects for the best model M1: Fixed effects of feedback from

Coeffic	χ^2^	df	P values
Feedback	158.60	2	<0.001
Stress	0.45	1	ns
Lack of Perseverance	0.001	1	ns
Lack of Premeditation	0.31	1	ns
Negative Urgency	0.39	1	ns
Time	5.87	1	0.02
Feedback X Negative Urgency	15.36	2	<0.001
Feedback X Lack of Perseverance	12.29	2	0.002
Feedback X Lack of Premeditation	1.72	2	ns
Feedback X Stress	2.07	2	ns
Stress X Negative Urgency	0.35	1	ns
Stress X Lack of Perseverance	1.05	1	ns
Stress X Lack of Premeditation	0.84	1	ns
Feedback X Negative Urgency X Stress	8.24	2	0.02
Feedback X Lack of Premeditation X Stress	4.15	2	ns
Feedback X Lack of Perseverance X Stress	26.28	2	<0.001

the previous trial (where feedback was coded as −1 for a loss, 0 for no feedback, and 1 for a win),

stress and impulsivity traits on subjects’ decisions to gamble (i.e., how much money subjects

gambled from €0 to €10). The significant effects are further explored in Table 5 below.

**Table 5:** Planned comparisons for the linear mixed-effects model M1: Fixed effects of feedback of the previous trial, stress and impulsivity traits on subjects’ decisions to gamble (i.e., how much money subjects gambled from €0 to €10).

Coeffic	Estimate (SE)	t value	P values
Intercept	8.99(2.38)	4.03	<0.001
Feedback(0)	−11.17(1.71)	−6.48	<0.001
Feedback(1)	−0.46(1.23)	−0.37	ns
Stress	−5.40(2.89)	−1.99	ns
Lack of Perseverance	−0.30(0.18)	−1.76	ns
Lack of Premeditation	−0.01(0.19)	0.03	ns
Negative Urgency	−0.11(0.14)	−0.87	ns
Time	−0.17(0.07)	−2.43	0.02
Feedback(0)X Negative Urgency	0.38(0.17)	2.19	0.02
Feedback(1)X Negative Urgency	−0.06(0.06)	−0.93	ns
Feedback(0)X Lack of Perseverance	0.76(0.15)	4.93	<0.001
Feedback(1)X Lack of Perseverance	0.09(0.09)	1.04	ns
Feedback(0)X Lack of Premeditation	0.13(0.14)	0.92	ns
Feedback(1)X Lack of Premeditation	−0.16(0.09)	−1.70	ns
Feedback(0)X Stress	8.51(2.30)	3.65	<0.001
Feedback(1)X Stress	−0.67(1.46)	−0.46	ns
Stress X Negative Urgency	−0.01(0.19)	−0.09	ns
Stress X Lack of Perseverance	0.50(0.23)	2.32	0.029
Stress X Lack of Premeditation	0.20(0.28)	0.78	ns
Feedback(0)X Negative Urgency X Stress	−0.01(0.20)	−0.03	ns
Feedback(1)X Negative Urgency X Stress	0.25(0.09)	2.77	0.005
Feedback(0)X Lack of Premeditation X Stress	−0.27(0.21)	−1.26	ns
Feedback(1)X Lack of Premeditation X Stress	0.15(0.13)	1.15	ns
Feedback(0)X Lack of Perseverance X Stress	−0.90(0.18)	−4.84	<0.001
Feedback(1)X Lack of Perseverance X Stress	−0.35(0.11)	−3.09	0.005

Feedback = feedback on the previous trial (1 = a win in the gambling task, −1 = a loss in the gambling task, 0 =.

## Discussion

The aim of the current study was to test the influence of individual differences in impulsivity traits on decision-making under ambiguity in a setting of laboratory-induced stress. In doing so, the study also served as a partial replication of a recent study^17^ by demonstrating for the first time the interplay between impulsivity traits, stress and decision-making under ambiguity. The results showed that a specific impulsivity facet, namely lack of perseverance, impacts the way that prior feedback is incorporated into decisions involving ambiguity in a condition of laboratory-induced stress. The discussion is divided in two parts. The first part pertains to the effects of stress on decisions under ambiguity, whereas the second part regards the moderating role of impulsivity traits in these effects.

The results showed that there was no general effect of stress on gambling decisions. Thus, the present study replicates many studies that have failed to show an effect of stress on gambling^64–66^. It is possible that betting behaviours in both conditions have been influenced by gambling-related biased cognition, more particularly, the gambler’s fallacy phenomenon^16^, defined as the tendency to respond to losses by increasing one’s bet, which reflects an inability to acknowledge the independence of turns. Subjects seemed unable to use a rational perspective (in the economic sense, see Rabin^15^), in which each new decision to gamble should be considered an independent event. In contrast, participants tended to use irrelevant prior experiences to guide their future choices to gamble, regardless of their stress levels. Thus, participants were more likely to gamble after receiving negative feedback (a loss in the previous trial) than after receiving positive feedback (a win in the previous trial), which is consistent with and replicates the findings of a recent study on the effects of acute stress on decision-making under ambiguity^17^.

These results are also in line with previous studies that used naturalistic gambling settings and reported increased risk taking following losses^67^. At least two potential explanations account for the behavioural results observed in the current study. First, individuals gambled more after experiencing a negative outcome in a potential attempt to recover the recent losses. Chasing losses, or betting more money after losses in an attempt to win back the money lost^68, 69^, is an indicator of misunderstanding gambling outcomes and irrational beliefs about the likelihood of winning^70^ or compromised inhibitory control^71^. Chasing losses is also known to occur within sessions among non-problem gamblers^72, 73^. Second, as the losing streak develops, the player progressively believes that the value of a significant win increasingly surpasses the negative value of a further loss^74^. According to the prospect theory value function, people experience diminishing marginal utility in both the gain and loss domains^75^. In the loss domain, this means that as a losing streak develops, people become more willing to gamble because a further negative outcome will not feel as bad as earlier negative outcomes. At the same time, a significant win would help reduce overall losses; therefore, it becomes increasingly attractive in comparison to refraining from gambling.

The present study shows that impulsivity traits play a crucial role in explaining the differential effect of stress in the relationship between prior feedback and choices made under ambiguity. More precisely, we found that acute stress appears to influence how feedback is incorporated into decisions involving ambiguity for subjects with lower perseverance. Individuals characterized by a lack of perseverance (in the stress condition) seems to display a behaviour that resembles the gambler’s fallacy phenomenon. This supports the idea that gambling can be self-serving (i.e., it can serve in reducing negative affective states generated by aversive events, e.g., losses in the previous trial) and encourage gambling perpetuation, as suggested by motivated reasoning models^76^. Actually, individuals with reduced perseverance are usually prone to boredom, have a limited sense of responsibility and present attentional difficulties^46^. It has been shown that they are less motivated to succeed in work or school^77^. Consequently, they may be easily distracted by exciting activities, such as gambling^78^. Furthermore, perseverance (like premeditation) is associated with the deliberation and self-discipline facets of conscientiousness^79^. Costa and colleagues^80^ conceptualized conscientiousness as a personality dimension involving both the need for achievement and commitment to work and moral scrupulousness or cautiousness. Thus, reduced perseverance is likely to increase the likelihood of displaying the gambler’s fallacy, as this impulsivity trait is related to less availability of general cognitive resources (including difficulties in inhibiting non-relevant thoughts or memories) and less conscientiousness of ongoing tasks^45, 46^. Additionally, according to the revised model of decision-making under objective risk conditions^9^, our results can reflect that acute stress combined with lower perseverance strengthened the impulsive system, which in turn may interfere with the controlled extraction of information, deliberation, or planning (reflective system), leading participants to act against their better knowledge (e.g., being more focused on negative feedback). More specifically, lack of perseverance reduced resistance to proactive interference in working memory, sustained attention, and set-shifting capacities^45–81^, which in turn might result in distractions and irrelevant thoughts that may interfere with project completion^41^. From such a perspective, lower perseverance, instead of premeditation (and negative urgency), can be considered a candidate for non-advantageous decision-making under ambiguity in conditions of stress. Our results extend the previous literature on feedback and stress (see Schiebener and Brand^9^ for a recent review) by showing the effects of personality traits in the relationship between acute stress and learning from previous negative feedback (loss). Disturbed learning from negative feedback might thus be the mechanism behind the more disadvantageous decisions reported among participants with lower perseverance in the stress condition.

Contrary to our hypotheses, lack of premeditation and negative urgency did not significantly influence how previous feedback is incorporated into decision-making under stress. The inconsistent effect of negative urgency might be related to the individual characteristics of participants in the current study (non-clinical participants). Generally, negative urgency is a risk factor for addictive behaviours^82^ rather than a potential predictor of non-pathological behaviour (e.g., at-risk or problem gambling). Thus, negative urgency could explain impaired performance on decision-making in a sample of pathological gamblers. With regard to lack of premeditation, it seems likely that this impulsivity facet is more related to a tendency to act without forethought in general, i.e. not in arousal or emotional contexts such as that in the current study. From such a perspective, lack of premeditation rather reflects poor deliberative processes (e.g., not taking into account all available information prior to a decision). This is in line with past research showing that lack of premeditation is related to difficulties in delaying rewards^83^ or that it predicts involvement in behaviours with tangible long-term negative outcomes for health, such as smoking^84^.

Some limitations of the study must be acknowledged. First, stress induction was assessed using subjective parameters of stressors^43^. Although several studies measured stress responses capturing subjective responses^37, 43^, physiological responses to stress should be incorporated into future studies^17, 85^. Second, the present study indicated that impulsivity traits interact with acute stress induction to predict decision-making under stress, yet other unconsidered personality variables that have been linked to decisions and stress responses (e.g., neuroticism^86^) should also be considered potential moderators in future research. Third, developmental differences in feedback processing^87^ and stress^88^ have been documented. A better understanding of how age, stress and decision-making interact is also warranted and deserves further investigation. Finally, we did not collect data on executive functions. Assessing the executive functions would be a valuable addition to the literature, as these functions are important for (i) developing a decision-making strategy, (ii) applying decision-making strategies, and (iii) revising decision-making strategies according to feedback^9^.

Despite these limitations, the present study is likely the first that clarified the moderating effects of impulsivity traits on the relationship between feedback processing and decision-making under stress. In particular, the current findings support the view that young healthy adults with reduced perseverance gamble more after experiencing a loss when they have experienced stress compared to those who did not experience stress. Although our results were obtained in non-clinical participants, they potentially open up avenues for interventions targeting decision-making-related pathologies, such as disordered gambling or substance abuse. Individuals with reduced perseverance may benefit from interventions designed to help them become familiar with important concepts related to gambling such as independent events, myths and facts and responsible choices^89^. Other interventions to consider are those that help individuals face and accept adverse emotions without relying on dysfunctional coping aiming to relieve negative affect in the short term without considering long-term consequences. Examples of such interventions might include mindfulness-based group interventions because they are able to reduce urgency and increase perseverance (the two impulsivity facets shown to influence gambling behaviours in the current study) in adolescents with emotion regulation difficulties^90^. These interventions can help gamblers transfer the locus of control for stress from external conditions (i.e., feedback-related gambling) to attentional resources and reduce salience and myopic focus on rewards (i.e., by undermining the intrinsic value that gamblers assign to potential wins)^91^.

## Electronic supplementary material

Supplemental materials
