# Supplementary material for: Impulsivity influences betting under stress in laboratory gambling
Source: Sci Rep. 2017 Sep 6;7:10668. doi: 10.1038/s41598-017-10745-9 (PMC5587697; doi:10.1038/s41598-017-10745-9)
Supplement: Supplementary file 1 — Supplemental materials [file 41598_2017_10745_MOESM1_ESM.pdf]

Impulsivity influences betting under stress in laboratory gambling

Natale Canale[1,*], Enrico Rubaltelli[1], Alessio Vieno[1], Andrea Pittarello[2], and Joël Billieux[3,4]

[1]Department of Developmental and Social Psychology, University of Padova, Padova, Italy
[2]University of Groningen, Groningen, The Netherlands
[3] Addictive and Compulsive Behaviour Lab (ACB-Lab). Institute for Health and Behaviour. Integrative Research Unit on Social and Individual Development (INSIDE), University of Luxembourg, Esch-sur-Alzette, Luxembourg
[4] Addiction Division, Department of Mental Health and Psychiatry, University Hospitals of Geneva, Geneva, Switzerland

*natale.canale@unipd.it

Supplemental materials

Learning model. As it is possible that multiple previous experiences shape subsequent decisions, we tested how subjects with higher impulsivity incorporate prior feedback (feedback from three trials back) into decision-making under stress. To test this alternative model, we recalculated the feedback on every trial, according to the type of feedback (or no feedback) received on the previous three trials. The three trials were then added to compute a unique score. For example, a subject received negative feedback on trial one (-1), negative feedback on trial two (-1), and no feedback on trial three (0). The value of feedback on three trials back is thus equal to -2. The feedback range is between -3 and +3. We then coded as -1 a sequence of two/three losses and as 1 a sequence of two/three wins. We used the same linear mixed-effects models (LMMs) presented in the manuscript. Results revealed that although there was a main effect of feedback on the previous three trials ($\chi^2(1)= 54.77$; $p<0.001$), contrasts indicate that subjects did not gamble significantly more after experiencing a sequence of losses than after experiencing a sequence of wins (B=-2.75, t = -1.03, p=0.30).

Amount won or lost in the previous trial as a continuous predictor. Results reveal that there was a three-way interaction among continuous feedback, condition, and feedback ($\chi^2(1) = 15.01$; $p<0.001$) (see table 1S). The test of the simple slopes revealed that the effect of continuous feedback in interaction with stress was significant only for a loss as previous feedback (B = -0.16, $\chi^2(1)=5.64$, p = .02), but not for a win (p = 0.14). Thus, individuals in the stress condition who lost more money in the previous trial gambled more after experiencing a loss than did those in the control condition who lost the same amount of money (figure 1S).

Money gambled for condition. Subjects gambled approximately the same amount of money in the control condition (M = €4.51, SD = 1.88) and in the stress condition [M = €4.17, SD = 1.62, t(58) = 0.95, p = .90] (independent samples t-test).

Choice rate in the lottery game. An examination of choice behaviour revealed that participants gambled at a higher rate in the stress condition (83%) than they did in the control condition (79%) [$\chi^2(1)=4.39$, p=0.036].

Coeffic	Estimate (SE)	T values	P values
Intercept	5.57(0.43)	12.88	<0.001
Continuous Feedback	0.09(0.04)	1.70	ns
Feedback(1)	-1.19(0.41)	-2.85	0.04
Time	-0.11(0.07)	-1.45	ns
Condition(1)	-1.93(0.55)	-3.45	<0.001
Continuous Feedback X Condition(1)	0.23(0.07)	3.35	<0.001
Continuous Feedback X Feedback(1)	-0.07(0.06)	-1.10	ns
Continuous Feedback X Condition	2.07(0.55)	3.35	<.001
Continuous Feedback X Condition X Feedback(1)	-0.35(0.09)	-3.87	<.001

Note:Feedback (-1= loss; 1=win; excluding no feedback trials); Continuous feedback (amount won or lost in the previous trial from €2 to €10)

Table 1S: Results of linear mixed-effects model: Fixed effects of feedback and stress on subjects' decisions to gamble (i.e., how much money subjects gambled from €0 to €10)

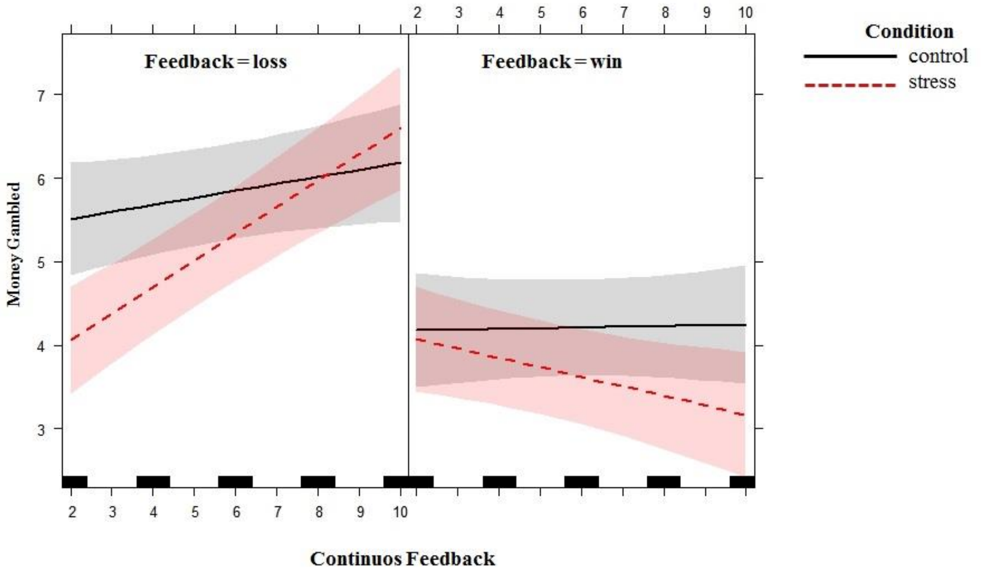

Note: Feedback: feedback on the previous trial (win = a win in the gambling task , loss = a loss in the gambling task; excluding no feedback trials); Continuous feedback (amount won or lost in the previous trial from €2 to €10); Confidence bands of 95% are presented in grey/red

Figure 3. Interaction plot for condition and feedback in relation to the amount of money gambled
